# Self-organization of conducting pathways explains electrical wave propagation in cardiac tissues with high fraction of non-conducting cells

**DOI:** 10.1371/journal.pcbi.1006597

**Published:** 2019-03-18

**Authors:** Nina Kudryashova, Aygul Nizamieva, Valeriya Tsvelaya, Alexander V. Panfilov, Konstantin I. Agladze

**Affiliations:** 1 Laboratory of Biophysics of Excitable Systems, Moscow Institute of Physics and Technology, Dolgoprudny, Russia; 2 Department of Physics and Astronomy, Ghent University, Ghent, Belgium; 3 Laboratory of Computational Biology and Medicine, Ural Federal University, Ekaterinburg, Russia; Mathematical Institute and the Institue for Biology, Leiden, NETHERLANDS

## Abstract

Cardiac fibrosis occurs in many forms of heart disease and is considered to be one of the main arrhythmogenic factors. Regions with a high density of fibroblasts are likely to cause blocks of wave propagation that give rise to dangerous cardiac arrhythmias. Therefore, studies of the wave propagation through these regions are very important, yet the precise mechanisms leading to arrhythmia formation in fibrotic cardiac tissue remain poorly understood. Particularly, it is not clear how wave propagation is organized at the cellular level, as experiments show that the regions with a high percentage of fibroblasts (65-75%) are still conducting electrical signals, whereas geometric analysis of randomly distributed conducting and non-conducting cells predicts connectivity loss at 40% at the most (percolation threshold). To address this question, we used a joint *in vitro*-*in silico* approach, which combined experiments in neonatal rat cardiac monolayers with morphological and electrophysiological computer simulations. We have shown that the main reason for sustainable wave propagation in highly fibrotic samples is the formation of a branching network of cardiomyocytes. We have successfully reproduced the morphology of conductive pathways in computer modelling, assuming that cardiomyocytes align their cytoskeletons to fuse into cardiac syncytium. The electrophysiological properties of the monolayers, such as conduction velocity, conduction blocks and wave fractionation, were reproduced as well. In a virtual cardiac tissue, we have also examined the wave propagation at the subcellular level, detected wavebreaks formation and its relation to the structure of fibrosis and, thus, analysed the processes leading to the onset of arrhythmias.

## Author summary

Cardiac arrhythmias are one of the major causes of death in the industrialized world. The most dangerous ones are often caused by the blocks of propagation of electrical signals. One of the common factors that contribute to the likelihood of these blocks, is a condition called cardiac fibrosis. In fibrosis, excitable cardiac tissue is partially replaced with the inexcitable and non-conducting connective tissue. The precise mechanisms leading to arrhythmia formation in fibrotic cardiac tissue remain poorly understood. Therefore, it is important to study wave propagation in fibrosis from cellular to tissue level. In this paper, we study tissues with high densities of non-conducting cells in experiments and computer simulations. We have observed a paradoxical ability of the tissue with extremely high portion of non-conducting cells (up to 75%) to conduct electrical signals and contract synchronously, whereas geometric analysis of randomly distributed cells predicted connectivity loss at 40% at the most. To explain this phenomenon, we have studied the patterns that cardiac cells form in the tissue and reproduced their self-organisation in a computer model. Our virtual model also took into account the polygonal shapes of the spreading cells and explained high arrhythmogenicity of fibrotic tissue.

## Introduction

The contraction of the heart is controlled by propagating waves of excitation. Abnormal regimes of the wave propagation may cause *cardiac arrhythmia*, asynchronous contractions of the heart and even lead to cardiac arrest and sudden cardiac death. Cardiac arrhythmias often originate from blocks of propagation [1]. In that case, the wave goes around the block, reenters the same inceptive region and, thus, forms a persistent rotational activity called *cardiac reentry*, which is one of the main mechanisms of lethal cardiac arrhythmias.

A normal heart has a complex structure, which is composed of the bundles of elongated cardiac cells. Apart from premier excitable cardiac cells, there are also inexcitable and non-conducting cells of connective tissue: *cardiac fibroblasts*. Their role is to maintain the structural integrity of the heart [2] and repair injuries [3]. Fibroblasts outnumber cardiomyocytes in a healthy human heart although occupying a much smaller total volume [2, 4]. However, many pathological conditions are associated with an excessive growth of the fibrous tissue, called *cardiac fibrosis*, which is, therefore, considered to be one of the major arrhythmogenic factors [5, 6].

The mechanisms of arrhythmia onset in fibrotic tissue remain poorly understood but generally believed to be associated with the increased probability of waveblock formation. It is a well-established fact that the presence of the non-conducting cells slows down wave propagation [7] and can completely block it if the non-conducting cells’ density is high. The critical density of non-conducting cells above which the conduction terminates is called *percolation threshold*. This concept originates from the percolation theory, and, by definition, it specifies the point of long-range connectivity loss/formation in random systems. Connectivity here refers to electrical synchronisation in the tissue, or, in other words, the ability to transmit electrical waves of excitation. Percolation threshold, i.e. the critical density of non-conducting cells which breaks long-range connectivity, plays an important role in arrhythmogenicity. It was shown that cardiac tissue is most susceptible to arrhythmias if the density of non-conducting cells is only slightly (∼2-3% [8]) below the percolation threshold. There are two main factors that may facilitate reentry formation. First, a large amount of non-conducting cells (acting as heterogeneities) increases the probability for waveblock formation [9]. Second, a high fraction of non-conducting cells creates a ‘maze’ that effectively lengthens the travel distance for the waves as they follow a longer zig-zag path [10] and, thus, provides sufficient room for the emerging reentrant loops. As a result, high density of non-conducting cells both facilitates the initiation and creates conditions for the existence of reentrant cycles, resulting in a highly arrhythmogenic substrate.

Up to now wave propagation failure was studied only in generic mathematical representations of heterogeneous cardiac tissue with each cell randomly chosen to be either conductive or non-conductive (see e.g. [9, 11]). In this kind of 2D computer models, the propagation of excitation failed at 40% of non-conducting cells [12], which is also within the range of values predicted by classic mathematical models (e.g. 37-44% of the area uncovered by conducting elongated ellipses with the shape similar to cardiomyocytes [13]). However, experimental measurements [14] indicate that wave propagation and synchronous contraction in 2D cardiac monolayers is observed for up to 75% percentage of non-conducting cells.

In this paper, we study the phenomenon of wave propagation in cardiac tissue with a high density of non-conducting cells using a joint *in vitro*-*in silico* approach. We performed experiments in 25 monolayers with various percentages of non-conducting cells and detected wave propagation to determine the percolation threshold. We have found that, indeed, the experimentally measured threshold (75% of the area covered by non-conducting cells) is substantially higher than what was predicted in conventional computer modelling (40% [12]) or classic mathematical models. Further morphological examination revealed that the key mechanism of conduction in highly heterogeneous tissue is likely to be tissue patterning. The cardiomyocytes were not located randomly but organised in a branching network that wired the whole sample.

Next, in order to explain cardiac network formation, we applied a virtual cardiac monolayer framework developed in [15], based on the Cellular Potts Models [16–18]. We proposed a hypothesis that such self-organisation occurs due to cytoskeletons’ alignment. Based on this hypothesis, we were able to obtain branching patterns, as well as reproduce the decrease in conduction velocity and wave percolation observed in experiments. We have further studied *in silico* the process of formation of the wavebreaks leading to reentry formation and analysed the tissue structures that caused them.

This paper is organized as follows. First, we describe the experiments conducted with the neonatal cell cultures. Second, we analyse the patterns and formulate the hypothesis on the mechanism of their formation. Third, we implement the hypothesis and reproduce pattern formation *in silico* in a Cellular Potts Model (CPM) of cardiac tissue [15]. Next, we compare electrical signal propagation in computer modelling and in experiments. Finally, we show a spontaneous formation of uni-directional blocks in the model resulting in reentry formation and locate and analyse the structures causing it.

## Results

### Experimental study of the percolation threshold in neonatal rat cardiac monolayers

We cultured neonatal rat ventricular cardiomyocytes mixed with cardiac fibroblasts in variable proportion (20-88% fibroblasts) and studied electrical activity in these monolayers using optical mapping.

**Fig 1:**
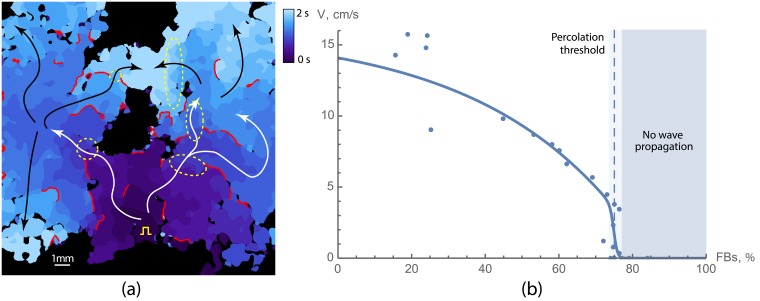
Wave propagation in neonatal rat cardiac monolayers with a large portion of non-conducting cells. (a) Activation map for a sample with 66% of non-conducting cells. Activation times are colour coded. Red lines show the regions where the wave was blocked. White and black arrows show the main propagation pathways. Yellow square pulse sign indicates the location of the stimulating electrode. Yellow dashed lines outline the areas of slow conduction. The original video (S1 Video) of the wave propagation is available at https://youtu.be/3aDmsT1pl3Y. (b) Velocity decay with the increase of the portion of non-conducting cells in samples. The percolation threshold is shown with the dashed line and was equal to 75 ± 2%.

In Fig 1a and in S1 Video the wave propagation in a sample with high fraction of non-conducting cells is shown (66%). In spite of high percentage of non-conducting cells, this sample is still conducting electrical waves, however the wave propagation pattern is complex. The wave originates from the stimulation point marked by a yellow spike at the bottom of the tissue and initially spreads in all directions. However, due to a large number of non-conducting cells, the wave is blocked at multiple sites. After a short delay (in areas outlined with dashed ellipses), the wave propagates further into the left and the right parts of the sample, and again spreads in various directions. This process repeats multiple times, resulting in a complex, fractionated wave pattern containing narrow pathways and some bulk excited regions. The conduction blocks in Fig 1a are shown in red, which are the places where the wave propagation was blocked and the wave had to go around. The main propagation paths are shown with white and black arrows. In this sample, the electrical wave propagation was still possible and long-range connectivity was still present, however the amount of non-conducting cells was close to the percolation threshold.

We have found that the percolation threshold for the neonatal rat cardiac monolayers was 75 ± 2% of non-conducting cells. We have also measured conduction velocities in the samples below the percolation threshold (Fig 1b). The measurements show that the velocity decreased when approaching the percolation threshold. In the samples with low level of non-conducting cells, the velocity was approximately 10-14 cm/s, and it decreased twofold in the samples with 70% non-conducting cells. The number of conduction blocks was higher in samples with high percentage of non-conducting cells. As a result, the mean conduction velocity decreased with the increase in a percentage of non-conducting cells.

**Fig 2:**
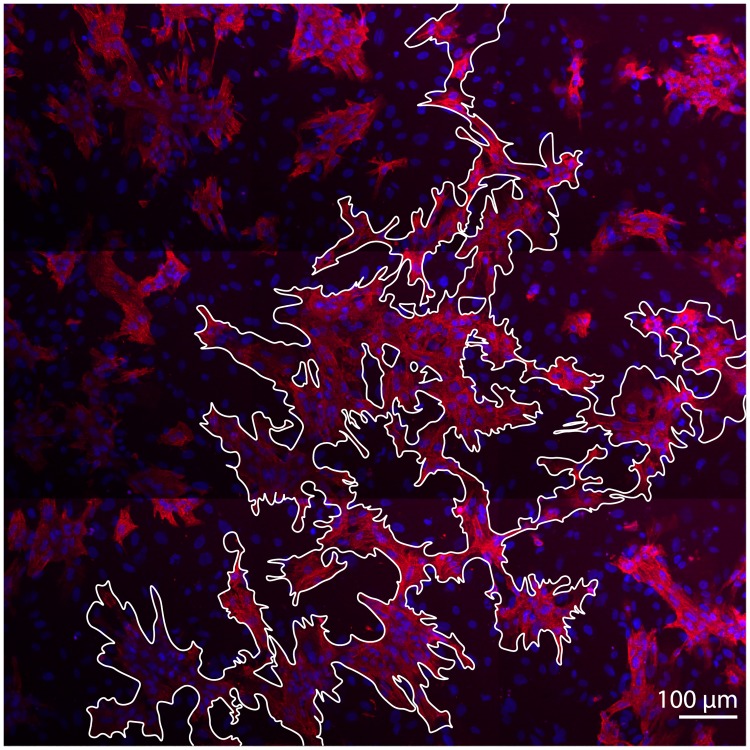
Conducting pathway in a monolayer of cardiac tissue with 31% of cardiomyocytes and 69% of non-conducting cells. The interconnected region is outlined in white. Cardiomyocytes are labeled with anti-*α*-actinin antibody and coloured in pink. Nuclei are shown in blue.

After optical mapping of the wave propagation, we have fixated the samples and studied their morphology using immunohistochemical labeling. We have found that the cardiomyocytes in the samples have formed connected networks capable of electrical wave propagation. In Fig 2 cardiomyocytes are shown in pink, and the cluster that they have formed is outlined with a white contour. The cardiomyocytes were organised in a branching structure that wired the whole sample. We have followed the pathway using a confocal microscope and it was possible to find long-range connectivity in the tissue. Therefore, we observed that the cardiomyocytes were organised in conduction pathways and assumed that there must be a mechanism responsible for their self-organisation.

### Developing a hypothesis for pattern formation

Pattern formation in cell populations during development was extensively studied using Cellular Potts Models [16, 17, 19], including many studies [20–22] of the branching structures similar to the one in Fig 2. Therefore, we have considered several existing hypotheses to explain the formation of the cardiac pathways.

**Fig 3:**
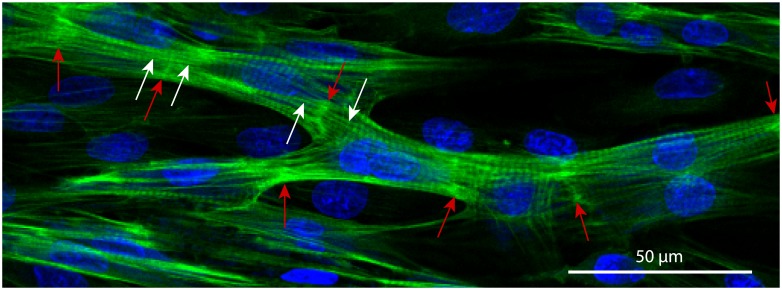
Cardiac syncytium in a 3-days culture of the neonatal rat cardiomyocytes. The cells have formed intercalated discs (ID, bright-green, highlighted with red arrows), aligned their cytoskeletons (the aligned strains on the both sides from ID are shown with white arrows) and formed a branching network. Nuclei are shown in blue (DAPI, labels DNA), and actin strands are shown in green (phalloidin, labels F-actin).

First, we suggested that differential adhesion together with cell elongation may be enough to explain the observed patterning. A similar system with autonomously elongating cells was successfully used to describe vasculogenesis [20]. However, we did not impose obligatory elongation on the cardiac cells, because they are not necessarily elongated according to our experimental observations (see Fig 3 below or S2 Video). Cardiomyocytes obtain their typical brick-like shape with the guidance of the extracellular matrix over the course of development. However, there is no evidence for any internal autonomous mechanism for elongation. In experiments on the glass, cells were not only bipolar but also tripolar and multipolar (see S2 Video). In our tissue growth model [15], which was accurate at replicating realistic cell shapes, this feature was reproduced with explicit introduction of the actin bundles. Cooperation between aligned actin bundles pulling in one direction shaped the cell more efficiently, which led to clustering of these virtual actin bundles and resulted in multipolar cell shapes. Similar approach was also previously used to describe the shapes of dendritic cells [23]. Therefore, since the elongation was not imposed, there was no mechanism forcing cardiomyocytes out of clusters to search for new connections.

Next, we considered various mechanisms that were previously used to explain similar branching patterns in angiogenesis. The main sources of instability in those models were chemotaxis and contact-inhibition [21]. The percolation in the networks formed due to sharp gradients of chemoattractants was also studied previously in a continuous model [22]. However, there was no evidence of any directed migration of cardiac cells and for type-specific contact-inhibition, similar to those that select a tip cell in a growing blood vessel. Therefore, we discarded this hypothesis either.

After trying several approaches used before, we have not found an existing model that could have been 1) applicable to the cardiac tissue and at the same time 2) could reproduce the experimentally observed branching structures. Finally, after careful analysis of our experimental preparation, we found that an essential feature of our structure was the alignment of the cytoskeletons in the neighbouring cells. This alignment of the actin fibres is clearly seen in our experimental preparations. In Fig 3 the bundle of neonatal rat cardiomyocytes is shown. The red arrows indicate the intercalated disks between the cells. The white arrows point at the actin bundles on the opposite sides of the intercalated disk, that smoothly continue one another.

### Formation of branching structures in a computer model

The precise biological mechanism of such alignment is unknown. However, in our view, it can be derived from a well-known property of actin cytoskeleton reinforcement in response to the external force [24]. Actin filaments, as well as the adaptors that link them, remodel under applied tension. In case of contact of two cells, the actin filaments are connected through adherens junctions, which transmit the tensile forces between the filaments of the neighbouring cells. It was shown that higher tension stabilises the whole complex [24]. The tension is maximal, if the actin filaments are aligned with each other, which gives a preference for intercellular alignment of the cytoskeletons.

Therefore, we have incorporated this mechanism into our Cellular Potts Model (CPM) of cardiac cells [15]. This model was already adjusted to reproduce characteristic shapes of the cardiac cells in virtual tissue model, and here we extended it with a new energy term, that corresponded to the alignment of actin bundles in the neighbouring cells.

**Fig 4:**
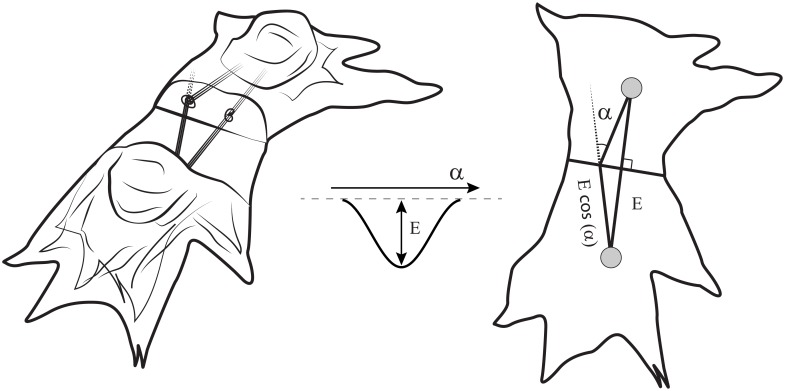
The schematic of the cell-to-cell interaction in a computational model. The energy term was assigned to every pair of connected actin bundles in coupled cells. This term depends on the angle between the bundles and reaches its minimal value when the bundles are aligned (*α* = 0). Left image shows quasi-3D schematic of the cells, middle image shows energy profile and right image shows a view from the top.

In our CPM model, cell-substrate adhesion sites, to which actin bundles are anchored, were represented as separate entities: specially labeled subcells of the lattice. If two adhesion sites of two cells came into contact, we established a new *connection* between them. The connection means, that an additional bond energy was applied to them. This energy depended on the angle between the linked actin bundles (see Fig 4). The minimum of the energy corresponded to smooth coupling between the bundles, or zero angle. In this case, the bond was the most stable, but couplings with the non-zero angle between the bundles had a tendency to break apart.

**Fig 5:**
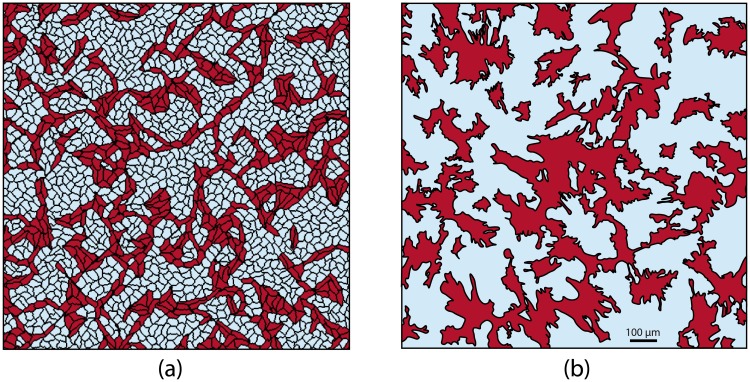
The branching pattern obtained in a computer model (a) compared to one observed in an experiment (b). **(a)** A virtual sample with 70% of non-conducting cells. **(b)** A segmented image of the experimental sample with 66% of non-conducting cells. The original image is shown in Fig 2.

With this new energy term that favours cytoskeletons alignment, the cardiomyocytes in simulations created branching patterns. In Fig 5a, a resulting simulated structure of the sample with 70% non-conducting cells is shown. We see that in this sample only 30% of cardiomyocytes were able to build a network, and even with such a high density of non-conducting cells, this network was fully interconnected. Our further studies showed that such interconnection was established for every sample with 30% of cardiomyocytes (*n* = 10) of 1 cm × 1 cm size, which is close to the size of the Petri dish used in our experiments. For samples with 28% of cardiomyocytes, the network was interconnected with 20% probability. We also see that the patterns in computer simulation (Fig 5a) and in experiment (Fig 5b) have similar features, such as long single-cell-wide connections that bridged the gaps between cell clusters, or isolated non-conducting clusters trapped within the main cardiac pathway. Therefore, using the hypothesis on cytoskeletons’ alignment allowed us to reproduce not only the percolation threshold, but also the main features of the pattern.

S2 Video shows the growth of the branching pattern, highlighting the large connected clusters of the cardiomyocytes by different colours. One can see from the video, that subtle movements of the cells (left) result in dramatic changes in connectivity (right). This emphasises the important role of the protrusions of the cells, which are taken into consideration in our model, in electrical signal propagation.

Using this model with cytoskeleton alignment, we have studied further wave excitation patterns and the percolation threshold for electrical waves in the system.

### Wave propagation in virtual cardiac tissue monolayers

We have reproduced the experiments from Fig 1a *in silico* using Majumder et al. [25] model for neonatal rat ventricular cardiomyocytes.

**Fig 6:**
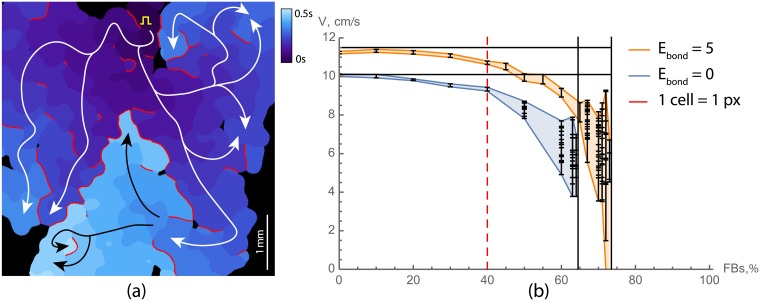
Wave propagation in virtual cardiac monolayers with a large portion of non-conducting cells. **(a)** Activation map for a sample with 70% of non-conducting cells. Activation times are colour coded. Red lines show the regions where the wave was blocked. White and black arrows are showing the main propagation pathways. Yellow square pulse sign indicates the location of the stimulating electrode. **(b)** Velocity as a function of the density of non-conducting cells. Orange lines represent velocities in virtual samples with cytoskeletons alignment (*E*_*bond*_ = 5), and blue lines without (*E*_*bond*_ = 0). Red dashed lines shows the percolation threshold for a simple model where each cell is represented by a point in a square lattice. Waves propagation in samples with branching patterns (orange) was failing in part of the samples starting from 71% and not possible in all of the samples for ≥75%. For each density, 10 samples with the size of 5 mm × 5 mm were tested. For each sample the mean velocity and its standard deviation are shown (mean ± SD). The original video (S3 Video) of the wave propagation is available at https://youtu.be/elvOvBRwnEM.

Fig 6a and S3 Video show wave propagation in a virtual sample with 70% non-conducting cells. The wave propagation pattern is complex and similar to one observed in experiment (see Fig 1a). One can see, that the number of propagation blocks per unit area is similar to that of the experimental activation pattern, and the trajectories of the waves share the same features. Note, that the spatial scale of the simulated activation map is slightly smaller than those of the experimental one.

The percolation threshold in virtual cardiac monolayers was equal to 71.5 ± 1.5% of non-conducting fibroblasts, meaning that the samples with such level of fibroblasts had 50% probability of percolation. In our simulations, 100% of the samples (*n* = 10) with 70% fibroblasts were interconnected, whereas samples with more than 73% fibroblasts (*n* = 10) were never conducting. For 72% fibroblasts 20% of the samples were functional and for 71% fibroblasts, those were 80%. The conduction velocity dependence on the density of fibroblasts is shown in Fig 6b. For each simulated sample we have indicated the mean value of the velocity, and the standard deviation of the velocity distribution was shown with error bars. One can see, that the dependency of the velocity on the fibroblasts’ density is similar to one measured in experiments (see Fig 1b). Closer to the percolation threshold the fluctuations in conduction velocities amplified due to the stochastic nature of the percolation block. Moreover, variations in conduction velocities within one sample were very high in that range, because of a large number of conduction blocks.

Thus, the percolation threshold in our simulations with *E*_*bond*_ = 5.0 was only slightly lower (≈ 72%) than in experiment (75%). However, both values are much higher than the predictions of the models with random cells distribution (40%).

Fig 6b also shows the percolation threshold in a computer model without cytoskeleton alignment (*E*_*bond*_ = 0, blue), which was equal to 64%. It is still higher than the threshold for randomly distributed cells (40%) since some clustering of the cells takes place. The reason for this clustering is that the adhesion between FB is slightly stronger than between cardiomyocytes or between cells of different types (*J*_*FB*−*FB*_ = 500 vs. *J*_*CM*−*CM*_ = *J*_*CM*−*FB*_ = 700). Such a difference is required to reproduce the difference in cell shapes correctly: all surface energies of the fibroblasts have to be lower than those of cardiomyocytes to reproduce dynamic fluctuations of their boundaries. The model in this study inherits some parameters from our previous paper [15], however it was reparameterized to ensure long-term stability of the model (on the scale of 20’000-50’000 MCS). We have first tried to enhance differential adhesion, but discovered that the differential adhesion alone was not capable of reproducing the branching pattern observed in experiments (as was discussed above). Thus, differential adhesion was tuned down as much as possible (*J*_*CM*−*CM*_ set to be equal to *J*_*CM*−*FB*_), preserving the desired cell shapes and volumes. Nonetheless, the resulting parameters cause some clustering even for *E*_*bond*_ = 0, which, in turn, raises the percolation threshold from 40% up to 64%. These data show to which extent the accurate representation of cell shapes and clustering of the cells of different types affect the percolation threshold (+24% compared to a simple square lattice model), and how much it is affected by cytoskeletons’ alignment (extra +8%).

### Optimal value of cytoskeleton coupling energy

**Fig 7:**
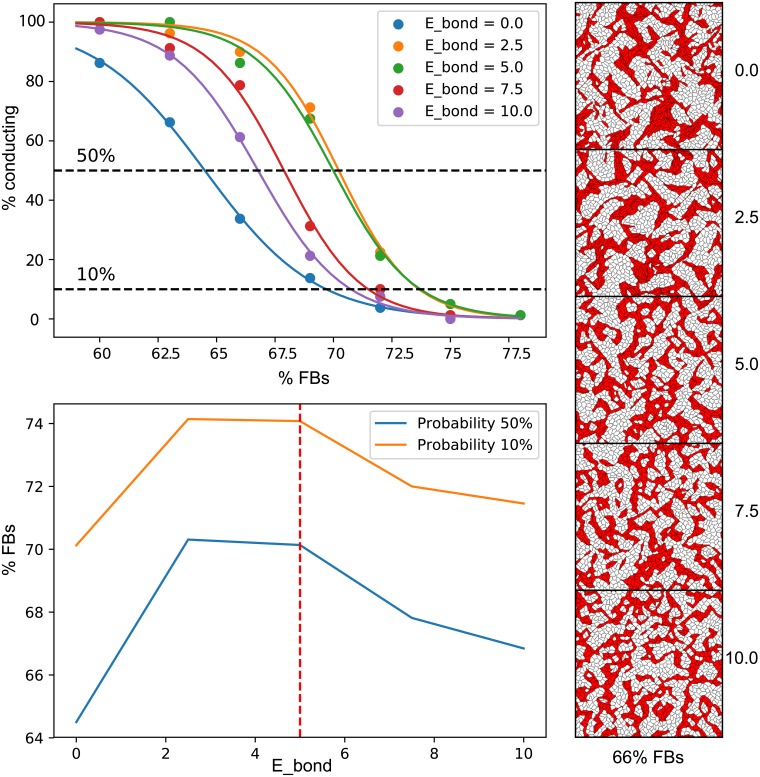
Optimal value of *E*_*bond*_. The top-left panel shows the probability of full connectivity in the sample (5 x 5 mm samples, connected from top to bottom and from left to right) depending on the percentage of non-conducting cells. Values of *E*_*bond*_ are shown with different colours. Each point corresponds to the average probability in 40 generated samples. The data was fitted with sigmoid functions. The bottom-left panel shows the percentages of non-conducting cells that correspond to 50% and 10% probability of connected network formation in a sample depending on the value of *E*_*bond*_. These plots show, that there exists an optimal value of *E*_*bond*_ that provides the highest chance of network formation. On the right, examples of connected networks with 66% non-conducting cells and different values of *E*_*bond*_ are shown. Cardiomyocytes are shown in red, and non-conducting fibroblasts in white.

We have studied various values of *E*_*bond*_ and have found that there is an optimal range of values that provides the highest percolation threshold. One can see in Fig 7 that both low (*E*_*bond*_ = 0.0) and high (*E*_*bond*_ > 7.5) cytoskeletons’ coupling energies result in the low percolation thresholds. When the coupling is weak, the cardiomyocytes do not form strong connections between each other and form clusters rather than networks (see top-right pattern). When the coupling is strong, the cardiomyocytes form very strong connections instead, which effectively “freeze” these cells in place and do not allow any motility, which is essential for network formation (see bottom-right pattern). Therefore, intermediate values of the *E*_*bond*_ are optimal for network formation, as they favour cytoskeleton alignment sufficiently, yet without imposing too much restriction on cells’ motility.

We have chosen *E*_*bond*_ = 5.0 for this study, since it belongs to the optimal range of values and, at the same time, reproduces the most qualitative features of the experimental samples. One can see in the middle-right image in Fig 7, that there are some fibroblasts (white cells) trapped in the clusters of cardiomyocytes (red cells). It is an important characteristic feature of the pattern, which is also present in our experimental samples (see right-hand image in Fig 5).

In Fig 7, we have shown two definitions of the percolation threshold corresponding to 10% and 50% probability of successful network formation. The 50% is typically used in theoretical studies [8], however here we compare the probability corresponding to 10% with the experiment. The reason for this is that it is not possible to carefully assess the probability of network formation in experiments. There are many other factors that may cause the propagation failure in cell culture and large statistics is needed to find the exact value corresponding to 50% probability of conducting network formation. Therefore, we have recorded all successfully conducting samples including those that belong to a vicinity of the percolation threshold and were still, by chance, conducting. Therefore, we suggest that 10% probability threshold provides a practical estimation of the highest percentage of non-conducting cells which we are likely to witness electrically connected, and thus this value is better for comparison with the experiment.

### The uni-directional block and spontaneous reentry onset observed in virtual cardiac monolayers with high portion of non-conducting cells

**Fig 8:**
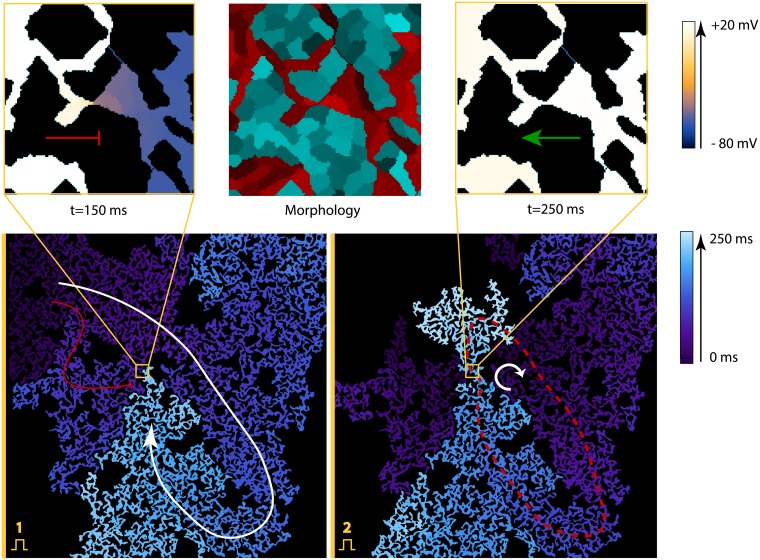
Spontaneous formation of the structure, that produces a uni-directional block, which results in reentry formation. The virtual sample had 29% of cardiomyocytes. Images in the top row show the insets of the place, where a uni-directional block has occurred: the central image shows its morphology and images on the sides show wave propagation in different directions. The voltage in these images is colour coded. In the central image, cardiomyocytes are represented with red tints and non-conducting fibroblasts with cyan. The bottom images show the activation maps in the whole sample. The bottom-left image shows the uni-directional block after the first stimulus, which was applied on the left boundary. The bottom-right image shows the reentry formation after the second stimulus. The activation times are colour coded. The arrows show the main wave paths. The red dashed line represents the reentry cycle. The original video (S4 Video) of the wave propagation is available at https://youtu.be/6LZorTUcJdk.

We have shown that in virtual tissues a spontaneous onset of reentry can occur. It was observed in samples with a high level of non-conducting cells. Fig 8 shows such process for a sample with 70% non-conducting cells. We see that after the first stimulus applied to the left border of the sample (shown in yellow), the wave propagation was blocked at the lower part of the sample, but it pursued through the upper part. After reaching the right border, the wave turned around and entered the bottom region. However, this first wave was blocked in the middle of the sample, as the tissue in the upper part of the sample have not been recovered yet. The wave from the second stimulus, applied at the same site, has followed the same path and formed a sustained circulation along the path shown with the red dashed line in Fig 8.

Detailed analysis of the structure revealed that formation of the reentry here is solely due to specific structure which is shown in the middle (inside the yellow square), which acts as an area of uni-directional block (or “diode”): the waves can propagate from right to left, but not in the opposite direction. The diode is formed by two cell clusters that barely touch each other. These cell clusters have slightly different areas adjacent to this connection. Therefore, if the wave propagates from left to right (from a smaller cluster to a larger one), then the small cell cluster does not produce enough current and can not depolarise a bigger one. The transmembrane potential in the largest cluster raises (which is shown in pink colour), but not enough for the sodium channels to open. This effect is called *source-sink mismatch* [1]. When the source (a smaller cluster) is insufficient compared to the sink (a larger inactive cell cluster), the wave propagation is blocked. This effect was observed in chemical systems [26] and later in cardiac monolayers [27].

In a sample in Fig 8, the “diode” could initiate a reentry if the sample is stimulated from the left or from the top with a high frequency (4 Hz or more).

We have performed studies in 6 large samples with 70% of non-conducting cells. All of these samples had 2-5 areas of the uni-directional block and many bi-directional blocks, but only one of these samples was arrhythmogenic. Thus, in addition to “diodes”, some extra geometrical conditions are required. The precise conditions are to be studied in the future, however, they are related to the presence of the long conducting circuits in the tissue. In fact, in Fig 8 we see that apart from the diode, there is also a loop, which is shown with a red dashed curve in the bottom-right image. The diode is a part of this loop, however, the loop is large enough to account for recovery of the bottom part of the tissue after one rotation. The samples with a higher density of non-conducting cells were even less likely to have long circuits, thus we did not observe sustained reentry there. We concluded, that reentry formation requires not only uni-directional blocks but also long circuits, and the densities of non-conducting cells slightly below the percolation threshold are the most arrhythmogenic ones. This result was previously shown for random cell distributions, that reentry is most likely to occur 5-10% below the percolation threshold [8]. The principle holds true in our model, but quantitatively the densities of non-conducting cells are different.

Our model shows, that the areas of the uni-directional block can be naturally formed during tissue growth. Every time one spreading cell comes in contact with the other cell cluster, there is a chance that this connection will be asymmetric. It may explain the fact that reentries are frequently observed in the experimental setups with neonatal cardiac monolayers.

## Discussion

We observed paradoxical electrical wave propagation in samples with up to 73–75% of non-conducting cells instead of mathematically predicted 40% for randomly distributed cells. We have shown both *in vitro* and *in silico*, that electrical wave propagation was possible due to the formation of the conduction pathways that rewired the whole monolayer. We have proved the existence of this branching network with immunohistochemical images. We have measured the conduction velocity, which decreased with the increase of the portion of non-conducting cells in the monolayer in a similar way in experimental and computational studies.

To explain the formation of the pathways, we have considered several existing hypotheses based on differential adhesion, cells elongation and directed migration. However, all of them were drawn inapplicable to the cardiac tissue. As a result, we developed and proposed a new hypothesis, based on cytoskeleton alignment. Assuming that cardiomyocytes align their cytoskeletons to fuse into cardiac syncytium, the morphology of conductive pathways was successfully reproduced in computer modelling. The virtually generated monolayers were then used for the studies of the electrical wave propagation, and experimentally observed velocity decay and high percolation threshold were reproduced as well.

The proposed hypothesis of cytoskeletons alignment has never been considered before. This mechanism, however, has some similarities to *diffusion limited aggregation* (DLA): a process in which random-walking particles form fractal aggregates. In our model, a cell ‘sticks’ to the growing pattern, but, unlike DLA, our cells also align cytoskeleton’s orientation with the pattern. Such alignment actually takes place as a part of syncytium formation, when cardiomyocytes fuse and align their cytoskeletons. Once a cell aligns cytoskeleton with its neighbours, this structure maturates and fixes the cell in place. In some sense, this process causes contact inhibition only between cardiomyocytes and not between cardiomyocytes and non-cardiomyocyte cells. Such type-specific contact inhibition results in the branching structure like one shown in angiogenesis [21].

Formation of conduction pathways and complex texture of the tissue affects arrhythmogenicity, and may also be important for arrhythmia treatment. For example, it was shown that texture of cardiac tissue at subcellular level can substantially affect the propagation of external current during defibrillation [28, 29]. It would also be interesting to quantify the excitation patterns in terms of the number of re-entrant sources and wavefront complexity [30]. Therefore, it will be interesting to perform a similar study for the textures generated with our model.

There are several limitations of the methods used in this study. First of all, we have conducted experiments with cell cultures, which are different from the real cardiac tissue. It would be interesting, yet more complicated, to study patterning of the real 3D cardiac tissue. However, the mechanisms of patterning that we discovered here are likely to be universal and might be applicable to the real cardiac development as well. Second, fibrosis is a more complex condition than just excessive growth of fibroblasts. It also involves collagen deposition that insulates the cardiac fibers from one another and is also considered to increase arrhythmogenicity. In this study, we did not take the extracellular matrix into account, but it would be also interesting to measure its effect on the percolation threshold in the future studies. Third, recent studies suggest that there are other conducting cell types in the heart, such as myofibroblasts [31] and macrophages [32, 33]. The fraction of these other cell types in the heart does not exceed 5-7% [34, 35], and, moreover, their amount in ventricular cell population, used in this study, is negligible [32]. However, presence of such cells in other cell populations may affect the percolation threshold. Finally, percolation depends on the size of the sample and scales with this size. For random systems, the scaling laws are known. In our case, we did not consider scaling and used similar size of the samples *in silico* as in the experiment. However, it would be interesting to study scaling of the percolation threshold and see how the size can affect the probability of wavebreak formation.

We conclude, that the cardiomyocytes in heterogeneous tissues with high portion of non-conducting cells can form a connected network and allow electrical signal propagation in monolayers containing up to 75% of non-conducting cells.

## Methods

### Ethics statement

All studies conformed to the Guide for the Care and Use of Laboratory Animals, published by the United States National Institutes of Health (Publication No. 85-23, revised 1996) and approved by the Moscow Institute of Physics and Technology Life Science Center Provisional Animal Care and Research Procedures Committee, Protocol #A2-2012-09-02.

### Experimental samples preparation

#### Neonatal cardiac cell isolation

In this study, we used existing two-day isolation protocol from Worthington-Biochem (http://www.worthingtonbiochem.com/NCIS/default.html). Briefly, hearts were extracted from rat pups (Rattus norvegicus, Sprague Dawley breed), aged 1–4 days, and immediately placed in Ca^2+^ and Mg^2+^ –free Hank’s Balanced Solution (Gibco, USA, 14180046) on ice. Only the tissue of the ventricles was isolated, providing that about 50-60% of the initial heart mass was cut off, which included the sinoatrial node, the atria, and the antro-ventricular node. The isolated ventricles were minced to small pieces and then left at 4°C temperature overnight for trypsinization. On the second day, cells were placed into collagenase solution (2.25 μg/mL, Gibco, USA, 17101015) and stirred for an hour at 37°C temperature.

To obtain a range of different cardiomyocytes to non-myocytes ratios in specimens, the following technique was used. First, suspension with the cells was placed into T75 flask for an hour for pre-plating. Then, the cells were counted with trypan blue (Gibco, USA, 15250061) and the concentration of the cells was adjusted to 10^6^ cells/ml. The cells were mixed in suspension with preliminary grown fibroblasts culture. The fibroblasts were obtained from previous cardiac cells isolations and grown in T75 flasks in DMEM (Gibco, USA, 11960) with 5% of FBS (foetal bovine serum, Gibco, USA, 10100147). They were detached by adding 0.25% trypsin-EDTA solution (Gibco, USA, 25200056), and then counted with trypan blue, and their concentration was also adjusted to 10^6^ cells/ml. The suspensions with isolated cells and fibroblasts culture were mixed in different proportions with 10% step to obtain different ratios of cardiomyocytes. Control samples seeded with 100% isolated cells (containing ≈ 70-80% cardiomyocytes) solution and 100% fibroblasts culture solution were seeded. It is worth noting that final percentage of cardiomyocytes present in growing culture differs from the one mixed in a suspension before seeding. Therefore, in this study we always refer to the final cell counts in grown samples (see Immunohistochemical staining below).

The obtained solutions with different cardiomyocytes to non-cardiomyocytes ratios were seeded in concentration about 2.5 ⋅ 10^5^ cells/cm^2^ on the cover slips covered with fibronectin (0.16 mg/ml, Gibco, USA, 33016015). The samples were cultivated in DMEM culture medium with 10% of FBS for the first 24 hours and then the media was changed to DMEM with 5% of FBS. After 3–5 days of cultivation, the monolayers were used for optical mapping and morphometrical studies. In the process of samples preparation fibroblasts were not activated. The stimulation of the samples was carried out strictly using an electrode.

#### Optical mapping

To monitor activity and record the excitation patterns, the 3- to 5-day-old monolayers were loaded with the Ca^2+^-sensitive indicator Fluo-4-AM (Molecular Probes, USA, F14201). After staining, the medium was exchanged with Tyrode’s solution (Sigma-Aldrich Co., USA, T2145-10L) and kept at 37°C temperature during the observations. The excitation waves were monitored with a high-speed imaging setup (Olympus MVX-10 Macro-View fluorescent microscope equipped with high-speed Andor EM-CCD Camera 897-U at 68 fps).

#### Velocity measurements

To standardize the velocity measurements, all samples were stimulated with platinum point electrode and referral ring electrode. The parameters of the stimulus were: 10 ms duration of the stimulus, 1 Hz frequency and 5V voltage. The stimulation frequency was always the same, which is important to avoid frequency-dependent changes in conduction velocity and action potential duration.

All videos were processed with ImageJ software. To measure the average velocity of excitation wave propagation in a specimen, especially the ones with low concentration of cardiomyocytes and thereby fluctuating velocity values, no less than 10 space intervals for each sample were selected, such that each interval was perpendicular to the wavefront. The velocity was measured for each interval and the mean value and standard deviation were calculated.

#### Immunohistochemical staining

Immediately after the optical mapping, the monolayers were fixated with 5% PFA solution (paraformaldehyde powder, 95%, Sigma-Aldrich, USA, 158127-100G), and nuclear staining was performed with DAPI (VECTASHIELD Mounting Medium with DAPI, Vector, USA, Cat. No. H-1200). In our work, we used anti-*α*-actinin (Sigma-Aldrich, USA, A7811)and Secondary Antibody with Alexa fluor 594(A-11020, Life Technologies) for CM-specific labelling, Alexa Fluor 488 phalloidin (Molecular Probes, USA, A12379) for F-actin non-specific staining and DAPI for labelling cell DNA. Pictures were taken with an inverted fluorescence microscope (Axio Imager with ApoTome optical sectioning module, Zeiss). Immunofluorescent staining of the CMs was performed with the use of secondary and primary antibodies according to a previously described protocol (http://www.abcam.com/protocols/immunocytochemistry-immunofluorescence-protocol). Signals from each fluorescent label were recorded in a corresponding wavelength range. Channels were then pseudo-coloured and merged together.

#### Cell counting

Cells were counted in confocal immunostained images using ImageJ. Nuclei (stained with DAPI) were segmented, and the cells positive on *α*-actinin were classified as cardiomyocytes. All the other cells were classified as “non-conductive”, since the majority of this cell population are the fibroblasts [34]. The total area covered by *α*-actinin positive cells was measured as well. For each sample, at least 2 confocal images covering 2.5 × 2.5 mm^2^ were processed. For nuclei separation, a standard morphological transformation (Distance Transform Watershed with distance option Borgefors (3, 4)) was used, which is the part of the MorphoLibJ library in Fiji ImageJ (https://imagej.net/Distance_Transform_Watershed). Cell counts were verified with cytofluorometer (Accuri C6, BD Biosciences, USA). The cells were stained with common for both cell types F-actin and specific cardiomyocytes marker *α*-actinin.

### Mathematical model for cardiac tissue growth

In this study, we have used a mathematical model that was previously developed for cardiac monolayers formation [15]. It is based on the Cellular Potts Model (CPM) formalism, which describes cells as the domains of the regular lattice and assigns energy to the system of cells to describe their growth and motility. In our previous work [15], we have selected the main features of the cardiac cells (such as area, number of protrusions, etc.) and parametrised our model to reproduce the cell shapes. In this study, we extended the model with a new energy term responsible for syncytium formation.

The evolution of the cardiac monolayer is described by the Hamiltonian:

$$H=H_{\text{adhesive}}+H_{\text{elastic}}+H_{\text{protr}}+H_{\text{nuclei}}+H_{\text{junctions}},$$ (1)

where *H*_adhesive_ + *H*_elastic_ is the basic CPM model and *H*_protr_ is the term describing the protrusion dynamics of the cardiac cells, which produces a characteristic polygonal shapes of these cells. *H*_nuclei_ corresponds to higher rigidity of the nuclei compared to the cell body. Finally, *H*_junctions_ is a new term, that describes the stability of adherens junctions and alignment of the cytoskeletons of the neighbouring cells.

The core feature of the model of cardiac cells is the explicit representation of cell attachments as the labelled subcells of the lattice. They are first assigned when the cell expands, and can be destroyed with a certain penalty, if, for example, the cell is stretched due to its movement. The number of attachments per cell is limited by the amount of actin present in the cytoplasm, which is also reflected in the model. If the maximal number of attachments is reached, new attachments do not form.

The spreading of virtual cells is shown in S5 Video. One of the important features of our approach, is that elongation of the cells is not imposed, but rather emerges due to interactions with the environment. Cardiac cells in a real heart are indeed elongated, as they are guided by the extracellular matrix, whereas in a Petri dish cells can spread in any direction with no preference. However, this does not result in a circular shape due to the limited number of attachment sites where spreading occurs. Instead of explicit declaration of elongation, we suggested that virtual cells have only a small (≈10) number of well-developed mature attachment sites. Moreover, these sites in a model tend to cluster spontaneously (see S5 Video), as the actin strands pull the cell more efficiently acting together, rather than separately. Therefore, these clustered attachment sites turn cell shapes into bipolar, tripolar or multipolar states. The bipolar state is the most stable one for isolated cells, however tripolar cells are also relatively common. This correctly represents the population of cells observed in experiments.

*H*_protr_ is an energy term, that decreases with the distance from the centre of mass of the cell, and which is applied only to these labeled attachment sites. As a result, the attachment sites spread out and reproduce characteristic polygonal shape of the cardiac cells. Here, in this model, we omit the details of the spreading process, which involves polymerization and depolymerisation of the actin, attachment/detachment, etc., but we mimic the overall dynamics of the protrusions. Also, we assume, that for every attachment site a corresponding actin bundle exists, which stretches from the attachment site towards the proximity of the nuclei.

If two attachment sites in the neighbouring cells come into contact, the bond between these attachment sites can be formed. In the algorithm, this bond appears if one attachment site attempts to move over the other. In such case, instead of copying of the subcell, the connection establishes. A new energy term *H*_junctions_ applies to the subcells, which are involved in a newly established junction. This term determines the stability of the cell-to-cell junction and depends on the angle between the actin bundles, associated with the attachment sites involved (see Fig 4). It is determined as follows:

$$H_{\text{junctions}}=\sum_{\begin{array}{c} \vec{i},\vec{j}\\ \text{labeled}\;\text{junction} \end{array}}E_{bond}\left(1-\text{cos}\left(\alpha_{i,j}\right)\right),$$

where *α*_*i*,*j*_ (shown in Fig 4) is the angle between two cytoskeleton bundles of two neighbouring cells which ends at points *i* and *j* labelled as the parts of the junction. The wider is the angle, the less stable is the junction. Therefore, the junctions with continuous actin bundles on both sides persist, but the kinked bundles tend to lose the connection. As a result, the actin bundles of the neighbouring cells tend to align and stay in the aligned state.

The parameters of the model used in simulations (see Table 1) in this paper were adjusted to compensate the additional energy term *H*_junctions_. The most of them are close to the parameters used in our original paper [15]. The value of the new energy term was set to *E*_*bond*_ = 5.0, which provides enough stability for the junctions to maintain the branching structure but at the same time not too much stability to allow cells to search for possible new connections. Addition of *H*_junctions_ effectively increased the adhesion between cardiomyocytes. Therefore, the differential adhesion was toned down in a model to allow cardiomyocytes to migrate randomly before they maturate and stick to the pattern. In this study, we have changed type-specific adhesion coefficients *J*_*cell*−*cell*_. Our choice of the parameters was guided by the desired balance between branching and clustering: 1) lower adhesion energy (*J*) to the cells of the same type increased the size of unstructured, irregularly shaped clusters; 2) on the other hand, high adhesion energy forced cells to migrate out of clusters. We have chosen neutral values of *J*_*CM*−*CM*_ = *J*_*CM*−*FB*_, which meant that cardiomyocytes had no preference in neighbours. Their energy was equal for being surrounded with either other cardiomyocytes, or with the non-conducting cells. The non-conducting cells had a slight tendency to cluster (*J*_*FB* − *FB*_ < *J*_*CM* − *FB*_). This choice of parameter allowed us to qualitatively reproduce the patterns observed in experiments (see Figs 2 and 5).

**Table 1:** Parameters of the morphological model used in stimulations. CM—cardiomyocytes, FB— fibroblasts.

Parameter	Units	CM	FB
Temperature T	1.0	1.0	
*G*_N_	mm	100.0	20.0
*V*_*t*_	⋅10^3^*μ*m^2^	0.9	0.8
λ	mm^−4^	60.0	20.0
*P*_detach_	mm^−1^	11.0	10.0
*J*_Cell-MD_		600.0	275.0
*J*_Cell-Cell_		700.0	500.0
*J*_CM-FB_		700.0	
*L*_MAX_	*μ*m	40.0	40.0
*N*_protr_ (fixed)		14	8
*E*_bond_		5.0	-
Sample dim.	mm × mm	4.8 × 4.8	
Simulation time	MCS	50000	
Number of cells	1	162 × 162	

The code used in this study is available on Github: https://github.com/NinelK/VCT. Examples 3 and 4 in the repository correspond to formation of cardiac pathways with or without cytoskeletons alignment.

### Simulations of wave propagation

The structure of the virtual samples was first generated with the Cellular Potts Model. After the cells were grown and statistical characteristics of the cells and clusters stopped changing (50’000 MCS), the resulting lattice was converted into a matrix of coupling coefficients for electrophysiological studies. The electrical coupling between non-conducting cells and any other cells was set to zero, coupling of the lattice points within the cells was set to *D*_*in*_, and, finally, coupling between cardiomyocytes was set to either *D*_*L*_ or *D*_*T*_ depending on the orientation of the cells’ virtual cytoskeletons. The methodology was already described in detail in our previous paper [15] and the ratio between coefficients was adjusted to match the anisotropy of the wave propagation in aligned cells (*D*_*in*_ = 100 × *D*_*L*_, *D*_*T*_ ≪ *D*_*L*_ and negligible). The magnitude of the coupling coefficients was slightly adjusted (*D*_*in*_ = 0.4 *cm*^2^/*s*, *D*_*L*_ = 0.4 × 10^−2^ *cm*^2^/*s*, *D*_*T*_ = 0 *cm*^2^/*s*) to reproduce experimentally measured maximal conduction velocity in the control samples with low portion of non-conducting cells. The same coefficients were then used for all simulations.

#### Geometrical connectivity testing

We have used two methods to test the electrical connectivity of the samples in this study. First, we have simulated the whole process of wave propagation as described above. However, simulation of the detailed model required significant computational resources and, therefore, was not suitable for the studies where large statistics was needed (such as variation of *E*_*bond*_ in Fig 7).

The second method was based on purely morphological characteristics of the generated cardiac monolayers. Importantly, we had to account for the fact, that excitation waves have front-curvature dependent properties, and, as a result, can not pass through the narrow pathways in the pattern. The critical width of such pathway between large cell clusters is approximately equal to the *nucleation radius*: the minimal radius of the depolarised circle in the tissue that can stimulate a wave. This one, in turn, can be estimated as *R* = *D*_*end*_/*V* = (0.004*cm*^2^/*s*)/(14*cm*/*s*) = 3*μm*, where *D*_*end*_ is the intercellular coupling and *V* is the conduction velocity. This corresponds to only 1 step in our discrete grid.

In the second method, we have eroded the pattern prior to analysis, to eliminate the narrow pathways in the pattern which are impermeable for the electrical waves. Level of erosion was adjusted to match the nucleation radius, and as a result was set to 1 iteration of binary erosion. This removed all pathways of 2px and less in width. The 2px, or 2 lattice points, in our simulations were equal to 2 × 2.5*μm* = 5*μm* ≈ 2 × *R*.

We compared two methods for a subset of the textures and the resulting thresholds were closely matched. Higher and lower levels of erosion were also tested and proved to be inadequate. This suggests that selected level of erosion removed those and only those narrow pathways, which were impermeable for electrical waves, and, thus, the second method is valid as an alternative computationally-efficient measurement of the percolation threshold.

## Supporting information

S1 Video Raw optical recording of the wave propagation in a neonatal rat cardiac monolayers with with 66% of non-conducting cells. This video is also available at https://youtu.be/3aDmsT1pl3Y.
(MP4)

S2 Video Growth of the branching pattern. The left part shows cardiomyocytes in red, and non-conducting cells in cyan, and the right part highlights connected clusters with different colours. The red cluster is the one that was growing from the centre and eventually wired the whole sample. Video can be also accessed at https://youtu.be/s9V86BFcMQY.
(MP4)

S3 Video Raw output of the simulation of the wave propagation in a sample with 70% of non-conducting cells. This video is also available at https://youtu.be/lw4p7cen0u4.
(MP4)

S4 Video Spontaneous formation of the structure, that produces a uni-directional block, which results in reentry formation. The virtual sample had 29% of cardiomyocytes. Left panels show wave propagation from bottom to top, and right panel in the opposite direction. Two bottom rows show enlarged insets of the place, where a uni-directional block has occurred. The video is also available at https://youtu.be/6LZorTUcJdk.
(MP4)

S5 Video Visualisation of spontaneous symmetry breakup and polarization of the cells. The video is also available at https://youtu.be/RatnaeS7N2M.
(MP4)
